# Biocontrol Potential of Rhizosphere Bacteria Against *Fusarium* Root Rot in Cowpea: Suppression of Mycelial Growth and Conidial Germination

**DOI:** 10.3390/biology14080921

**Published:** 2025-07-23

**Authors:** Qinghua Zhu, Yixuan Ma, Tong Zhang, Weirong Liu, Songbai Zhang, Yue Chen, Di Peng, Xin Zhang

**Affiliations:** 1 MARA Key Laboratory of Sustainable Crop Production in the Middle Reaches of the Yangtze River, Yangtze University, Jingzhou 434025, China; 2 Hunan Plant Protection Institute, Hunan Academy of Agricultural Sciences, Changsha 410125, China; 3 Yuelushan Laboratory, Changsha 410082, China; 4 Hunan Institute of Microbiology, Hunan Academy of Agricultural Sciences, Changsha 410009, China

**Keywords:** cowpea, *Fusarium* spp., microbial antagonists, rhizobacteria, conidial germination

## Abstract

The cultivation of cowpea (*Vigna unguiculata*), a vital vegetable crop, faces significant threats from *Fusarium* spp.-induced root rot. In this study, three fungal pathogens (*Fusarium falciforme* HKFf, *Fusarium incarnatum* HKFi, and *Fusarium oxysporum* HKFo) were isolated from symptomatic cowpea plants, and we screened 90 rhizobacteria from healthy rhizospheres using six culture media. Among these pathogens, *Priestia megaterium* TSA-10E showed a notable suppression of *F. oxysporum* HKFo (63.21%), *F. incarnatum* HKFi (55.16%), and *F. falciforme* HKFf (50.93%). In addition, *Bacillus cereus* KB-6 inhibited the mycelial growth of *F. incarnatum* HKFi and *F. oxysporum* HKFo by 42.39% and 47.93%, respectively. Critically, cell-free filtrates from *P. megaterium* TSA-10E and *B. cereus* KB-6 cultures reduced conidial germination in *F. oxysporum* HKFo and *F. incarnatum* HKFi, highlighting their role in disrupting the early infection stages. In greenhouse trials, TSA-10E and KB-6 reduced disease severity by 48.7% and 40.4%, respectively, with treated plants maintaining healthy growth while untreated controls succumbed to wilting. Broad-spectrum assays revealed that *B. subtilis* TSA-6E and *P. megaterium* TSA-10E were potent antagonists against both economic and grain crop pathogens. These findings underscore the potential of rhizobacteria as sustainable biocontrol agents for managing root rot disease caused by *Fusarium* spp. in cowpea cultivation.

## Simple Summary

Cowpea, a vital crop for food security in many regions, is threatened by root rot disease caused by harmful fungi, leading to severe crop losses and reliance on chemical fungicides that harm the environment. This study aimed to find eco-friendly solutions by examining bacteria that naturally live near cowpea roots. We discovered that certain bacteria, especially *Priestia megaterium* TSA-10E and *Bacillus subtilis* KB-6, could block the growth of disease-causing fungi by 63.21% and 47.93%, respectively. These bacteria also produce natural compounds that stop fungal spores—the “seeds” fungi use to spread—from sprouting, reducing spore germination by up to 50%. Greenhouse trials demonstrated that TSA-10E and KB-6 treatments reduced disease severity by 48.7% and 40.4%, respectively, with treated plants maintaining healthy seedlings and intact roots, while untreated plants collapsed and died. By combining bacteria that work broadly against many pathogens with those targeting specific fungi, we propose a sustainable way to protect cowpea crops without chemicals. These findings offer farmers a safer, nature-based strategy to control root rot, improving crop yields while protecting soil health and reducing pollution. This research supports global efforts to promote sustainable farming and food security, particularly in regions where cowpeas are a dietary staple.

## 1. Introduction

Cowpea (*Vigna unguiculata*), a globally cultivated legume, is a vital source of plant-based protein and contributes to soil nitrogen fixation, underpinning its importance in food security and sustainable agriculture [1]. However, cowpea production is severely threatened by root rot disease, which manifests as leaf wilting, root necrosis, and vascular discoloration, ultimately leading to plant death [2,3]. Pathogens implicated in cowpea root rot include *Fusarium* spp., *Ectophoma multirostrata*, *Berkeleyomyces rouxiae*, *Phytophthora vignae*, and *Macrophomina phaseolina* [4,5,6]. Among these pathogens, *Fusarium oxysporum* is a soil-borne pathogen capable of causing yield losses ranging from 30% to 100% [5], which can be exacerbated by its resilient chlamydospores that persist in the soil for years.

Traditional reliance on synthetic fungicides to manage *Fusarium* spp. has led to pathogen resistance and environmental toxicity, driving the need for sustainable alternatives such as biological control agents (BCAs) [7]. For instance, *Ralstonia solanacearum* can be inhibited by a rhizobacteria strain, *B. amyloliquefaciens* known as JK6 [8]. More recently, microbial antagonists, such as *Bacillus* spp. and *Trichoderma* spp., have shown promise for inhibiting pathogens that cause root rot disease in other plants, including *Fusarium* spp. [9,10]. Therefore, the biocontrol performed by BCAs against cowpea root rot disease may be feasible and effective.

Rhizosphere-derived BCAs, including *Bacillus* and *Pseudomonas* spp., have demonstrated efficacy through mechanisms such as antibiotic production, niche competition, and induced systemic resistance [8,11]. Notably, suppressing conidial germination—a critical phase in fungal pathogenesis—has emerged as a key biocontrol strategy. For instance, lipopeptides from *B. subtilis* ZD01 significantly inhibit *Alternaria solani* conidial germination by disrupting spore integrity [12], underscoring the potential of microbial metabolites to target early infection stages.

The rhizosphere microbiome, which is essential for plant health, is dynamically reshaped by pathogen invasion, with diseased plants exhibiting reduced microbial diversity compared to their healthy counterparts [13,14]. Leveraging this information, we isolated three *Fusarium* strains (*F. falciforme* HKFf, *F. incarnatum* HKFi, and *F. oxysporum* HKFo) from diseased cowpea plants and screened rhizobacteria from healthy rhizospheres for antagonistic potential. To elucidate mechanistic insights, we further evaluated the ability of bacterial metabolites (from *Priestia megaterium* TSA-10E and *Bacillus cereus* KB-6) to suppress conidial germination. This approach aims to advance the development of multifunctional BCAs for integrated disease management in cowpea cultivation.

## 2. Materials and Methods

### 2.1. Isolation and Identification of Fungal Pathogens

Fungal pathogens were isolated from cowpea plants exhibiting root rot symptoms in Haikou City, China. Tissue segments (5 mm) were excised from the junction between necrotic lesions and asymptomatic regions. After rinsing thoroughly under running tap water, the explants were surface-sterilized by sequential immersion in 70% ethanol (0.5–1 min) and 2% NaClO (2–3 min). This was followed by three rinses with sterile distilled water to remove residual sterilizing agents. The sterilized segments were aseptically transferred onto potato dextrose agar (PDA, Solarbio, Beijing) plates supplemented with ampicillin (50 µg/mL) to inhibit bacterial growth and incubated at 28 °C. Fungal hyphae emerging from the explants were subcultured onto fresh PDA plates for purification.

For molecular identification, the total genomic DNA was extracted using the CTAB method [15]. PCR was performed with universal primers ITS4 (5′-TCCTCCGCTTATTGATATGC-3′) and ITS5 (5′-GGAAGTAAAAGTCGTAACAAGG-3′) to target the ITS sequence [16]. The amplifications were performed in 25 μL reaction mixtures containing 1 μL of each primer (10 μmol/L), 12.5 μL of Premix Taq™ (Takara Bio, Beijing), and approximately 100 ng of the template genomic DNA. The thermal cycling conditions were as follows: initial denaturation at 95 °C for 5 min; 33 cycles of 95 °C (1 min), 56 °C (1 min), and 72 °C (1 min); and a final extension at 72 °C for 5 min. Molecular identification of *Fusarium* isolates was also performed using species-specific primers targeting the translation elongation factor (TEF) gene region. Four primer sets, TEF-1F (5′-CTTAACGTCGTCGTCATCG-3′)/TEF-2R (5′-CACTTGGTGGTGTCCATCTT-3′), TEF-1F/TEF-3R (5′-TTGTAGCCGACCTTCTTGAT-3′), EF-LF1 (5′-CCTTAACGTCGTCGTCATCG-3′)/EF-LR1 (5′-ACGGACTTGACTTCAGTGGT-3′), and EF-LF1/EF-LR2 (5′-GAGCTGCTCGTGGTGCATCT-3′), were employed [17]. PCR reactions were carried out as mentioned above. The amplification protocol consisted of initial denaturation at 94 °C for 2 min; 35 cycles of 94 °C for 30 s, 60 °C for 40 s, and 72 °C for 90 s; followed by a final extension at 72 °C for 5 min. The PCR products were sequenced, and sequence similarity was analyzed via BlastN against the GenBank database at the National Center for Biotechnology Information (NCBI).

### 2.2. Isolation and Identification of Rhizosphere Microbes

Rhizosphere soil samples (2 g) were collected from healthy cowpea plants in the same field. Microbial isolation was performed using a serial dilution plating technique on six distinct media: LB (Luria–Bertani medium), 0.1 × LB, KB (KingB medium), YG (yeast extract medium), R2A (Reasoner’s 2A agar), and TSA (Tryptic Soy Agar) (Table S1). Soil suspensions were prepared by vortexing the samples in 18 mL sterile physiological saline (0.9% *w*/*v* NaCl) for 40 min at 200 rpm and 28 °C, followed by dilution to 10^−4^–10^−7^. Aliquots (0.1 mL) of 10^−5^–10^−7^ dilutions were spread onto triplicate plates of each medium and incubated at 28 °C. Morphologically distinct colonies were selected and subcultured for purity.

Bacterial identification was based on 16S rRNA gene sequencing. Colony PCR was conducted using primers 27F (5′-AGAGTTTGATCCTGGCTCAG-3′) and 1378R (5′-CGGTGTGTACAAGGCCCGGGAACG-3′) [18] under the same conditions as mentioned above. Sequences were compared to the GenBank database at the National Center for Biotechnology Information (NCBI) via BlastN for taxonomic assignment.

### 2.3. Screening for Antagonistic Activity

To assess the antifungal activity of rhizosphere isolates, a dual-culture assay was used, which was modified from the method described by De Vrieze et al. [19]. Mycelial plugs (8 mm diameter) of the target pathogens were placed centrally on LB agar plates. Bacterial isolates were cultured in 5 × YEG (yeast glucose medium) broth (200 rpm, 30 °C, 48 h), and 40 µL of each culture was applied to four sterile filter paper discs (diameter: 5 mm). The discs were positioned 2 cm from the plate edge at 90° intervals. The plates were incubated at 28 °C for 3–6 days, and radial mycelial growth was measured. The inhibition rate was calculated as follows:

Inhibition rate (%) = [(A − B)/A] × 100

Here, A and B represent mycelial growth in the control and treatment groups, respectively. Experiments were performed in triplicate with three independent repetitions.

### 2.4. Broad-Spectrum Antifungal Activity Assay

To comprehensively evaluate the biocontrol potential of the selected rhizobacteria, we included a diverse range of plant pathogens in our screening. The selected bacterial isolates were tested against seven economically important pathogens affecting different crops: *Colletotrichum gloeosporioides*, *Sclerotium rolfsii*, *Fusarium solani*, *Magnaporthe oryzae*, *Exserohilum turcicum*, *Helminthosporium maydis*, *Sclerotinia sclerotiorum* (fungi), and *Phytophthora capsici* (Oomycete). This broad-spectrum approach was adopted for three key reasons: first, to assess whether the antagonistic activity was specific to *Fusarium* pathogens or whether it could extend to other phytopathogens; second, to identify potential multi-target biocontrol agents that could be applied across different cropping systems; and last but not the least, to evaluate the consistency of inhibitory effects across taxonomically diverse pathogens, which would indicate more robust biocontrol mechanisms. Antagonistic activity was evaluated using the filter paper disc method (Section 2.3), with inhibition rates quantified as described above. Experiments were performed in triplicate with three independent repetitions.

### 2.5. Conidial Germination Assay

Cell-free culture filtrates (CFCs) of *P*. *megaterium* TSA-10E and *B*. *cereus* KB-6 were prepared via centrifugation (8000 rpm, 10 min) and sterile filtration (0.22 µm). The conidia of *Fusarium oxysporum* HKFo and *Fusarium incarnatum* HKFi were harvested from 5×YEG cultures (28 °C, 72 h), filtered through Miracloth (Calbiochem, El Cajon, CA, USA), and adjusted to 5 × 10^4^ conidia/mL by a hemocytometer. The conidial suspension, which was treated with CFCs (10% *v*/*v* final concentration), was incubated at 28 °C for 5 h. Controls received 5×YEG medium. Germination rates were determined microscopically by counting ≥50 conidia per replicate. Three biological replicates were analyzed, each with three technical repeats.

### 2.6. In Planta Biological Control

The biocontrol efficacy of *P. megaterium* TSA-10E and *B. cereus* KB-6 against *F. oxysporum* HKFo and *F. incarnatum* HKFi was evaluated in greenhouse-grown cowpea (*Vigna unguiculata* cv. Changjiang 3). Seedlings germinated in peat-based substrate were transplanted into 9 cm diameter plastic pots containing a 3:1:1 peat-vermiculite-perlite mix, maintained at 25 °C under 12 h light/dark cycles with biweekly fertilization until the two-leaf stage. Prior to inoculation, roots were wounded near the stem base by puncturing with a sterile syringe needle (26-gauge) to a depth of 2–3 mm. Conidial suspensions of both pathogens were adjusted to 1 × 10^7^ conidia/mL, with four treatment groups established: pathogen control (CK1) receiving 10 mL mixed conidial suspension (5 mL per pathogen); TSA-10E group treated with pathogen suspension plus 10% *P. megaterium* filtrate; KB-6 group receiving pathogen suspension with 10% *B. cereus* filtrate; and negative control (CK2) administered pathogen suspension with 10% 5×YEG broth. Inoculated plants were kept in greenhouse at 25 °C. Each group contained at least 10 seedlings and was replicated three times. After 14 days, disease severity was scored on a 1–5 scale (1 = healthy, 5 = plant death) according to the grading standard of Rigert and Foster [20]. Disease index = Σ (number of diseased plants × representative series)/(total number of plants × highest representative level value) × 100. Control efficacy (%) = (control disease index − treatment disease index)/control disease index × 100%.

### 2.7. Statistical Analysis

All experiments were independently repeated three times, and the data were statistically analyzed using an ANOVA. The multiple Duncan test was used to compare the differences between mean values at a significance level of *p* < 0.05. All analyses were conducted using SPSS software (version 20.0; SPSS Inc., Chicago, IL, USA).

## 3. Results

### 3.1. Isolation and Molecular Identification of Pathogenic Fusarium Strains

Three morphologically distinct fungal strains were isolated from symptomatic cowpea tissues. Molecular identification via ITS and sequencing demonstrated >97% similarity with *Fusarium* species. Strain-level identification was achieved through BLASTN analysis with four species-specific primer pairs, confirming that the isolates as *Fusarium falciforme* HKFf, *F. incarnatum* HKFi, and *F. oxysporum* HKFo. The sequence similarity values are summarized in Table S2.

### 3.2. Isolation and Taxonomic Diversity of Rhizobacterial Strains

A total of ninety rhizobacterial isolates were obtained from six distinct culture media. 16S rRNA sequencing identified thirty-six non-redundant strains, most of which belonged to *Bacillus* spp. (34.4%, thirty-one isolates), followed by *Priestia* spp. (25.6%, twenty-three isolates) and *Neobacillus* spp. (11.1%, ten isolates). *Bacillus* exhibited the highest species diversity, comprising fourteen distinct strains (Table 1). Medium-specific isolation patterns were observed. The LB medium yielded thirteen strains, whereas YG and R2A each yielded eleven strains. The TSA medium yielded eight strains, and KB and 0.1×LB yielded seven and six strains, respectively. Notably, *Priestia megaterium* was recovered from all six media tested. In contrast, several strains exhibited medium specificity: *Bacillus bataviensis*, *Bacillus bingmayongensis*, *Bacillus safensis*, *Dyella thiooxydans*, *Paenibacillus septentrionalis*, and *Sinomonas atrocyanea* were exclusively isolated from R2A; *Bacillus pumilus* and *Bacillus subtilis* were unique to TSA; *Paenibacillus silvae*, *Neobacillus citreus*, and *Bacillus cereus* were found only in KB; and *Bacillus anthracis*, *Bacillus zanthoxyli*, *Heyndrickxia oleronia*, and *Rossellomorea marisflavi* were restricted to YG medium. Furthermore, with the exception of *P. megaterium*, all strains obtained from 0.1×LB medium showed strict medium specificity (Table S3).

### 3.3. Antagonistic Activity of Rhizosphere Isolates

**Figure 1:**
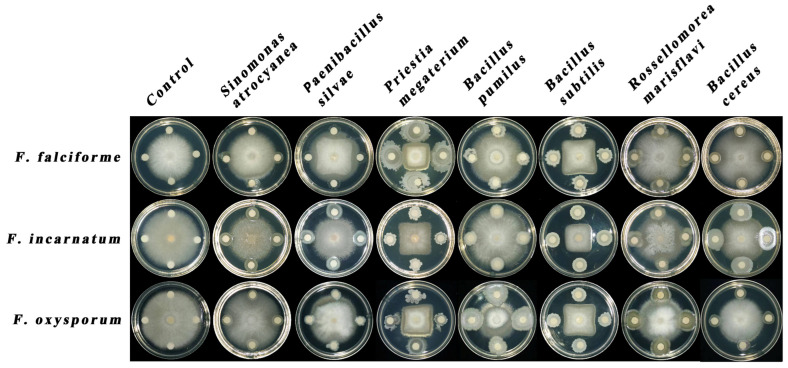
Antagonistic activity of rhizobacteria against cowpea root rot pathogens evaluated via a dual-culture assay. Mycelial plugs (8 mm diameter) of pathogenic fungi were placed centrally on LB agar plates, with four filter paper discs (5 mm diameter) loaded with 40 µL of bacterial culture (grown in the 5×YEG broth at 30 °C for 48 h) positioned 2 cm from the plate edge at 90° intervals. Control plates received 5×YEG medium instead of bacterial culture. All plates were incubated in a 28 °C incubator and cultured for three to six days.

Among the thirty-six rhizobacterial strains screened, seven (19.4%) exhibited antifungal activity against *Fusarium* pathogens, with their quantified inhibition rates presented in Table 2. Four strains—*P. megaterium* TSA-10E, *B. subtilis* TSA-6E, *B. cereus* KB-6, and *R. marisflavi* YG-2C—showed >40% inhibition of *F. incarnatum* HKFi and *F. oxysporum* HKFo. However, only *P. megaterium* (50.93%) and *B. subtilis* (46.27%) exceeded 40% inhibition against *F. falciforme* HKFf (Figure 1). *P. megaterium* TSA-10E demonstrated the highest overall antagonism, followed by *B. subtilis* TSA-6E. Against *F. incarnatum* HKFi and *F. oxysporum* HKFo, *B. cereus* KB-6 ranked third in terms of inhibitory efficacy, while *R. marisflavi* YG-2C ranked fourth. Notably, 42.9% (3/7) of the effective strains belonged to *Bacillus* spp., with *B. subtilis* TSA-6E and *B. cereus* KB-6 achieving ≥40% inhibition. In contrast, *S. atrocyanea* R2A-7 and *P. silvae* KB-5 exhibited <40% inhibition across all the pathogens. Additionally, the inhibition rates against *F. incarnatum* HKFi were consistently higher than those for *F. oxysporum* HKFo and *F. falciforme* HKFf.

### 3.4. Inhibition of Conidial Germination by TSA-10E and KB-6 Filtrates

While *P. megaterium* TSA-10E, *B. subtilis* TSA-6E, and *B. cereus* KB-6 showed strong antagonistic activity against *Fusarium* spp., we selected *P. megaterium* TSA-10E and *B. cereus* KB-6 for mechanistic studies. TSA-10E was prioritized due to its consistently high inhibition across *Fusarium* pathogens and the relative novelty of its biocontrol potential compared to the extensively studied *B. subtilis*, while KB-6 exhibited broader efficacy against key *Fusarium* strains compared to other isolates.

**Figure 2:**
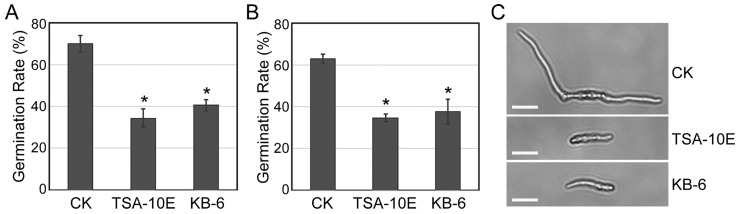
*P. megaterium* TSA-10E and *B. cereus* KB-6 cell-free filtrates inhibit the conidia germination of *F. oxysporum* HKFo (**A**) and *F. incarnatum* HKFi (**B**). Error bars represent the standard errors. The asterisks (*) represent significant differences compared with CK according to the multiple Duncan test (*p* < 0.05). (**C**) Morphological observations of the conidia of *F. oxysporum*. Bar = 10 μm.

To investigate their antifungal mechanisms, we evaluated the effect of cell-free filtrates from *P. megaterium* TSA-10E and *B. cereus* KB-6 cultures on conidial germination—a critical stage in *F. oxysporum* HKFo and *F. incarnatum* HKFi pathogenesis. Treatment with TSA-10E filtrates reduced *F. oxysporum* conidial germination by 50.9%, while KB-6 filtrates achieved 42.1% inhibition (Figure 2A,C). Similarly, *F. incarnatum* germination rates declined to 34.7% (TSA-10E) and 37.6% (KB-6) compared to untreated controls (Figure 2B). Microscopic examination revealed that in TSA-10E and KB-6 treated samples, the few conidia of *F. oxysporum* or *F. incarnatum* that managed to germinate produced markedly shorter germ tubes compared to the long, well-developed germ tubes observed in the controls (Figure 2C). These results demonstrate that both filtrates significantly disrupted early infection processes by suppressing conidial germination.

### 3.5. TSA-10E and KB-6 Can Effectively Control Cowpea Fusarium Root Rot in Greenhouse

**Figure 3:**
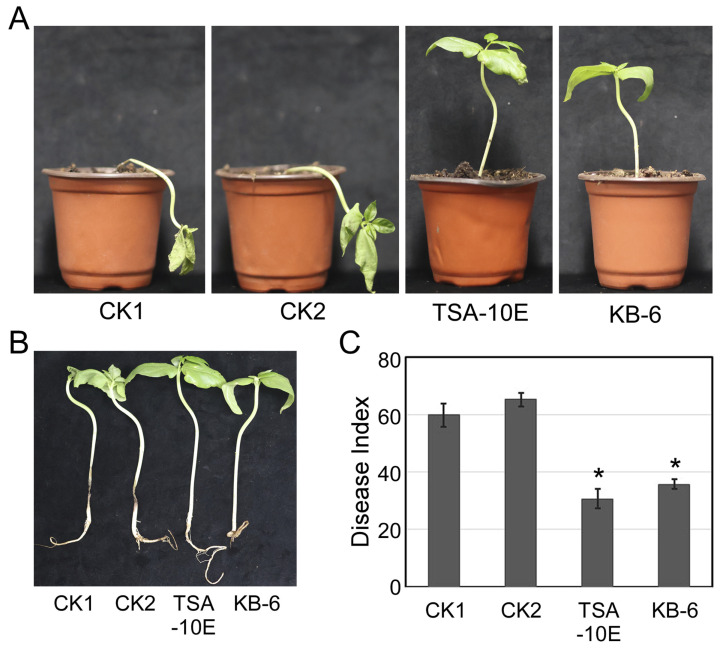
*P*. *megaterium* TSA-10E and *B*. *cereus* KB-6 suppress cowpea *Fusarium* root rot in greenhouse conditions. (**A**) Disease symptoms in cowpea seedlings. (**B**) The symptoms of cowpea roots. (**C**) The disease index of cowpea *Fusarium* root rot. Error bars represent the standard errors. The asterisks (*) represent significant differences from control by the multiple Duncan test (*p* < 0.05). Treatments: CK1 (*Fusarium* only), CK2 (*Fusarium*+medium), TSA-10E (*P. megaterium* TSA-10E treatment), and KB-6 (*B. cereus* KB-6 treatment).

Both *P. megaterium* TSA-10E and *B. cereus* KB-6 treatments provided effective protection against *Fusarium* root rot in cowpea plants. While control plants collapsed with severe wilting, treated plants maintained healthy and upright growth (Figure 3A). Root systems of treated plants showed minimal browning and rot, in contrast to the extensively decayed roots of controls (Figure 3B). Both treatments substantially decreased the disease index, with TSA-10E showing 48.7% control efficacy and KB-6 exhibiting 40.4% efficacy (Figure 3C). Notably, TSA-10E demonstrated superior disease suppression relative to KB-6. These results confirm the significant inhibitory effects of both bacterial strains against cowpea *Fusarium* root rot under greenhouse conditions.

### 3.6. Broad-Spectrum Antifungal Activity

**Figure 4:**
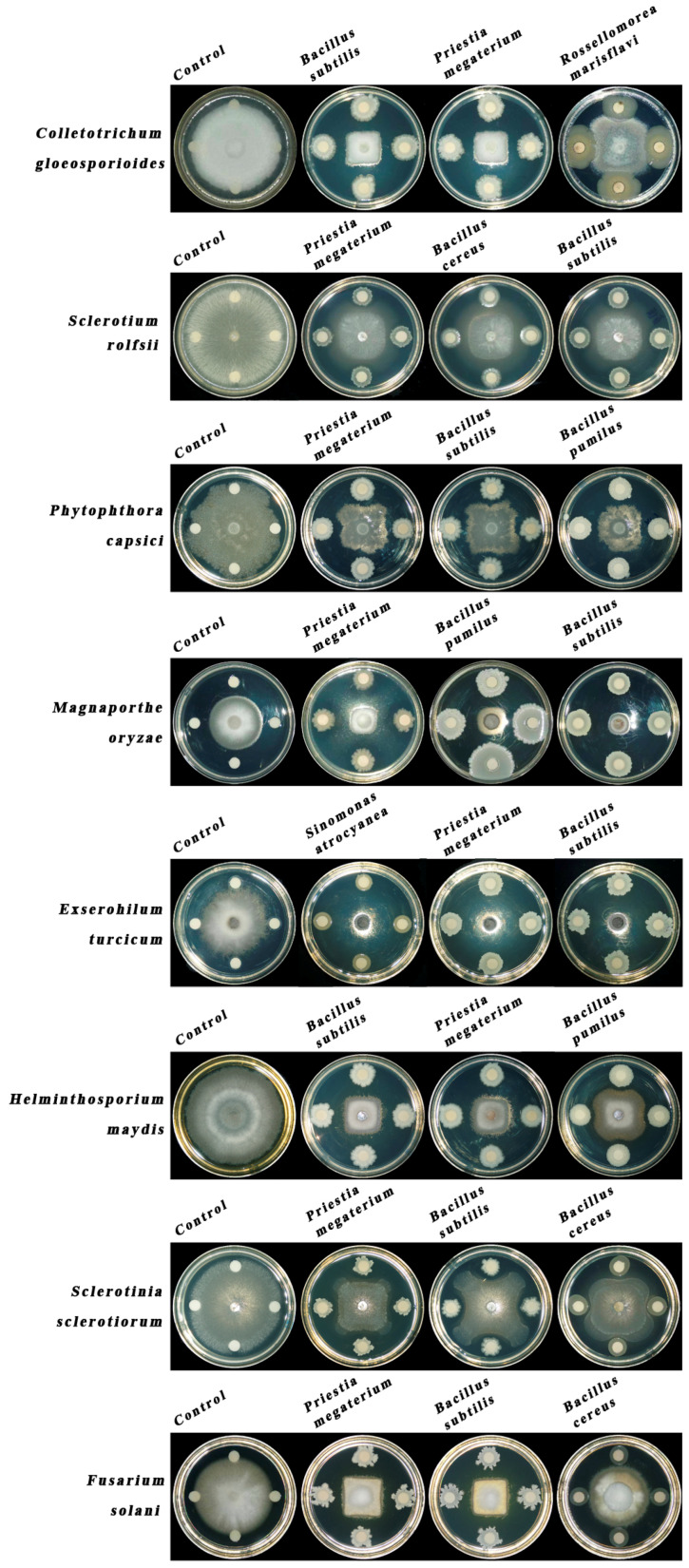
Antagonistic effect of rhizobacteria against the common pathogens that were selected, evaluated via a dual-culture assay. Control: pathogenic fungi with the 5×YEG medium on the LB medium. The plates were incubated in a 28 °C incubator and cultured for three to seven days.

The broad-spectrum antifungal potential of the seven selected rhizobacterial strains was evaluated against eight plant pathogens, including both grain crop pathogens (*Magnaporthe oryzae*, *Exserohilum turcicum*, and *Helminthosporium maydis*) and economic crop pathogens (*Colletotrichum gloeosporioides*, *Sclerotium rolfsii*, *Phytophthora capsici*, *Fusarium solani*, and *Sclerotinia sclerotiorum*). Among the tested strains, *B. subtilis* TSA-6E and *P. megaterium* TSA-10E exhibited consistent inhibitory activity across multiple pathogens (Table 3 and Figure 4). Against *C. gloeosporioides*, *B. subtilis* TSA-6E demonstrated the highest inhibition rate (77.76%), significantly surpassing *P. megaterium* TSA-10E (64.76%), *R. marisflavi* YG-2C (59.69%), and *B. cereus* KB-6 (55.90%). Similar trends were observed for *S. rolfsii*, with *B. subtilis* TSA-6E (48.32%) and *P. megaterium* TSA-10E (46.72%) showing superior suppression compared to *B. cereus* KB-6 (40.54%). Notably, *P. megaterium* TSA-10E and *B. subtilis* TSA-6E also displayed strong antagonism against *M. oryzae*, achieving inhibition rates of 75.91% and 73.73%, respectively, followed by *B. pumilus* TSA-1 (66.23%). In contrast, *S. atrocyanea* R2A-7 exhibited exceptional activity against *E. turcicum* (73.25%), marginally outperforming *P. megaterium* TSA-10E (72.76%) and *B. subtilis* TSA-6E (71.86%). However, none of the strains effectively inhibited *S. sclerotiorum* (<40% inhibition). For *F. solani*, both *B. subtilis* TSA-6E and *P. megaterium* TSA-10E achieved identical inhibition rates (53.18% and 52.29%), highlighting their broad-spectrum efficacy. These results underscore the differential inhibitory patterns among strains, with *B. subtilis* TSA-6E and *P. megaterium* TSA-10E emerging as promising candidates for controlling diverse phytopathogens.

## 4. Discussion

Rhizosphere-associated bacteria play a pivotal role in suppressing soil-borne pathogens through diverse mechanisms [21]. In this study, seven rhizobacterial isolates demonstrated antagonistic activity against fungal pathogens associated with cowpea root rot, with *B. subtilis* TSA-6E and *P. megaterium* TSA-10E exhibiting broad-spectrum efficacy. Notably, our findings extend to the inhibition of conidial germination by bacterial metabolites, a critical early-stage defence mechanism against fungal infection.

The isolation of thirty-six non-redundant strains from six media revealed distinct microbial preferences. The LB medium yielded the highest diversity (thirteen strains), likely due to its nutrient-rich composition, which supports diverse bacterial communities. Several strains exhibited medium specificity, such as *B. pumilus* and *B. subtilis* were exclusively isolated from TSA, while *B. cereus* was only found on the KB medium. These observations align with previous reports that medium composition selectively enriches specific microbial taxa by fulfilling their metabolic requirements [22].

The superior performance of *P. megaterium* TSA-10E against multiple pathogens (e.g., a 75.91% inhibition of *M. oryzae* and a 72.76% inhibition of *E. turcicum*) is indicative of its ability to deploy multifaceted biocontrol strategies. Such broad-spectrum activity may stem from synergistic mechanisms, including antibiotic production (e.g., lipopeptides), nutrient competition, and biofilm formation [11,23,24]. For instance, Zhang et al. [12] demonstrated that lipopeptides from *B. subtilis* ZD01 suppressed conidial germination in *Alternaria solani*, which is consistent with our observation that *P. megaterium* TSA-10E cell-free filtrates reduced *F. oxysporum* HKFo and *F. incarnatum* HKFi conidial germination by 50.9% and 44.8%, respectively (Figure 2). This finding represents an important advancement in understanding *P. megaterium* TSA-10E’s biocontrol potential, as previous studies have primarily focused on its broad-spectrum suppression of mycelial growth or mycotoxin production in various pathosystems. For example, while *P. megaterium* strains have been shown to produce antifungal lipopeptides like surfactin and iturin A against bacterial pathogens such as *Erwinia amylovora* [25], suppress *Fusarium verticillioides* mycotoxins through volatile organic compounds [26], or inhibit *Fusarium oxysporum* f. sp. ciceri [27], their direct effects on fungal spore germination remain unexplored. Our work provides the first evidence that *P. megaterium* TSA-10E can effectively disrupt the critical early infection stage of *Fusarium* pathogens by inhibiting conidial germination, a mechanism that has not been previously documented for this species.

Microscopic examination revealed that in addition to reducing germination rates, the *P. megaterium* TSA-10E and *B. cereus* KB-6 treatments caused notable morphological alterations in *F. oxysporum* HKFo and *F. incarnatum* HKFi conidia (Figure 2C). Compared to the control group, where conidia had normal, elongated germ tubes, treated conidia showed either germination inhibition or produced significantly shorter germ tubes. This laboratory observation directly translated to disease suppression in greenhouse conditions, where the same filtrates reduced *Fusarium* root rot severity by 48.7% (TSA-10E) and 40.4% (KB-6). The correlation between conidia inhibition and in planta protection suggests that disrupting early infection stages (germination and germ tube development) effectively prevents subsequent root colonization and disease establishment [28].

The significant reduction in conidial germination and germ tube length by *P. megaterium* TSA-10E and *B. cereus* KB-6 filtrates suggests that bacterial metabolites directly interfere with the early infection processes. Similar mechanisms have been reported for *Bacillus* spp., where antifungal compounds such as surfactin and iturin inhibit spore germination and hyphal elongation, and suppress watermelon *Fusarium* wilt [29,30]. The greenhouse results further support that this early-stage interference has functional consequences throughout the infection cycle, ultimately preserving plant health. The cell-free supernatants of the *Pseudomonas poae* strain CO were able to inhibit the spore germination and germ tube length of *Fusarium graminearum*, and exhibited diverse antifungal properties, such as the production of hydrolytic enzymes, siderophores, and lipopeptides [31]. These structural abnormalities suggest that the bacterial filtrates may interfere with the genetic and biochemical processes during germination, potentially through the action of antimicrobial metabolites. The combined evidence from microscopic observations and greenhouse protection assays suggests a potential mode-of-action sequence, where metabolite-mediated spore inhibition may lead to impaired germ tube formation, subsequently limiting root penetration and ultimately contributing to reduced disease symptoms. Our results reinforce the importance of metabolite-mediated inhibition as a key biocontrol trait, particularly against *Fusarium* spp., which rely heavily on conidia for host colonization. The consistency between in vitro and in planta outcomes underscores the translational potential of these findings for field applications.

While *S. atrocyanea* R2A-7 exhibited strain-specific inhibition (73.25% against *E. turcicum*), its limited efficacy against other pathogens underscores the value of combining broad-spectrum and specialized biocontrol agents. This phenomenon aligns with findings in other biocontrol systems. For instance, *Pseudomonas monsensis* H16 could inhibit *Alternaria brassicae YB43-2* and *Epicoccum sorghinum* YB53-2 but exhibit minimal activity against other phytopathogens [32]. Similarly to our observations with *S. atrocyanea* R2A-7, this narrow-spectrum activity suggests that these strains may have evolved specialized mechanisms targeting specific pathogens, possibly through the production of pathogen-specific antimicrobial compounds or interference with particular virulence factors. While narrow-spectrum agents lack versatility, their precision reduces off-target effects, making them ideal for integration into “polymicrobial” consortia. This “polymicrobial” strategy has garnered significant interest in recent years, although most of the research to date has primarily concentrated on blending well-established, commercially accessible microbial agents such as *Trichoderma* and *Bacillus/Pseudomonas* or mycorrhizal fungi and nitrogen-fixing bacteria [33,34,35,36]. Recent research has shown that two strains that have complementary modes of action are expected to be particularly efficient in their dual combination [19]. Rhizobacteria with broad-spectrum properties (*B. subtilis* TSA-10E, *P. megaterium* TSA-10E, and *B. cereus* KB-6) can be combined with rhizobacteria with more pathogen-specific antagonism (*S. atrocyanea* R2A-7), which could enhance disease control by targeting multiple infection pathways. This approach aligns with emerging trends in biocontrol research that emphasize functional redundancy and complementary action modes.

While this study identifies promising biocontrol candidates, further research is needed to characterize the active compounds in *P. megaterium* TSA-10E and *B. cereus* KB-6 filtrates and validate their stability under field conditions. Furthermore, elucidating the relative contributions of specific mechanisms such as antibiotic production and induced systemic resistance will refine strain selection for targeted applications.

## 5. Conclusions

This study demonstrates the potential of rhizosphere bacteria, particularly *P. megaterium* TSA-10E and *B. subtilis* TSA-6E, as eco-friendly biocontrol agents against *Fusarium*-induced root rot in cowpea. These strains exhibited dual antifungal mechanisms, suppressing both mycelial growth (>40%) and conidial germination (up to 50.9%) in key pathogens such as *F. oxysporum* HKFo and *F. incarnatum* HKFi. Greenhouse trials confirmed these effects under realistic conditions, with TSA-10E and KB-6 reducing disease severity by 48.7% and 40.4%, respectively, while preserving plant viability—demonstrating translational potential beyond in vitro systems. The broad-spectrum activity of *B. subtilis* and the novel efficacy of *P. megaterium* highlight their versatility in targeting multiple infection stages, offering a sustainable alternative to chemical fungicides. Furthermore, the inhibition of spore germination by bacterial metabolites (*P. megaterium* TSA-10E and *B*. *cereus* KB-6) underscores the role of secondary compounds in disrupting early fungal colonization, a mechanism now validated to correlate with whole-plant protection.

These findings not only expand the repertoire of biocontrol candidates but also support the development of polymicrobial formulations combining broad-spectrum and pathogen-specific strains. The consistency between laboratory and greenhouse results strengthens the case for field testing of these strains as integrated disease management solutions. Future studies should focus on characterizing the active metabolites, optimizing delivery methods, and validating these results under field conditions to ensure practical applicability. By bridging lab-based discoveries with agricultural needs, this study contributes to sustainable crop protection and food security in regions reliant on cowpea cultivation.

## Figures and Tables

**Table 1:** The rhizosphere microbes identified from different genera.

Genus	Total of Isolates	Total of Species	Species	Quantity
*Bacillus*	31	14	*Bacillus anthracis*	1
			*Bacillus aryabhattai*	11
			*Bacillus bataviensis*	1
			*Bacillus bingmayongensis*	1
			*Bacillus cereus*	2
			*Bacillus ferrooxidans*	3
			*Bacillus mycoides*	3
			*Bacillus niacini*	1
			*Bacillus pseudomycoides*	3
			*Bacillus pumilus*	1
			*Bacillus safensis*	1
			*Bacillus sporothermodurans*	1
			*Bacillus subtilis*	1
			*Bacillus zanthoxyli*	1
*Dyella*	1	1	*Dyella thiooxydans*	1
*Falsibacillus*	1	1	*Falsibacillus pallidus*	1
*Fictibacillus*	6	1	*Fictibacillus barbaricus*	6
*Gottfriedia*	1	1	*Gottfriedia acidiceleris*	1
*Heyndrickxi*	1	1	*Heyndrickxia oleronia*	1
*Neobacillus*	10	4	*Neobacillus citreus*	1
			*Neobacillus drentensis*	2
			*Neobacillus ginsengisoli*	6
			*Neobacillus niacini*	1
*Paenibacillus*	4	4	*Paenibacillus cellulositrophicus*	1
			*Paenibacillus pabuli*	1
			*Paenibacillus septentrionalis*	1
			*Paenibacillus silvae*	1
*Priestia*	23	2	*Priestia aryabhattai*	11
			*Priestia megaterium*	12
*Ralstonia*	1	1	*Ralstonia pickettii*	1
*Rossellomorea*	1	1	*Rossellomorea marisflavi*	1
*Sinomonas*	2	1	*Sinomonas atrocyanea*	2
*Streptomyces*	7	3	*Streptomyces anandii*	5
			*Streptomyces geysiriensis*	1
			*Streptomyces triostinicus*	1
*Trinickia*	1	1	*Trinickia diaoshuihuensis*	1

**Table 2:** Inhibition rate of three pathogens isolated by seven rhizobacteria.

Isolate	Species	Inhibition Rate (%)		
		*F. falciforme* HKFf	*F. incarnatum* HKFi	*F. oxysporum* HKFo
R2A-7	*Sinomonas atrocyanea*	21.88 ± 0.022 ^cd^	33.75 ± 0.007 ^de^	21.26 ± 0.017 ^e^
KB-5	*Paenibacillus silvae*	16.20 ± 0.055 ^d^	31.94 ± 0.042 ^e^	29.14 ± 0.009 ^d^
TSA-10E	*Priestia megaterium*	50.93 ± 0.029 ^a^	55.16 ± 0.001 ^ab^	63.21 ± 0.022 ^a^
TSA-1	*Bacillus pumilus*	21.12 ± 0.090 ^cd^	39.32 ± 0.008 ^cd^	36.73 ± 0.025 ^c^
TSA-6E	*Bacillus subtilis*	46.27 ± 0.035 ^a^	58.54 ± 0.001 ^a^	49.12 ± 0.034 ^b^
YG-2C	*Rossellomorea marisflavi*	33.51 ± 0.036 ^b^	43.10 ± 0.011 ^cd^	40.95 ± 0.029 ^c^
KB-6	*Bacillus cereus*	28.56 ± 0.021 ^bc^	47.93 ± 0.006 ^bc^	42.39 ± 0.015 ^bc^

Each value is the mean (±SE) of at least three replications. Values in columns followed by the same superscript letters indicate similar significance difference according to the multiple Duncan test (*p* < 0.05).

**Table 3:** The results of broad-spectrum antifungal property testing of seven selected rhizobacteria.

Isolate	Species	Inhibition Rate (%)							
		*C. gloeosporioides*	*S. rolfsii*	*P. capsici*	*M. oryzae*	*E. turcicum*	*H. maydis*	*S. sclerotiorum*	*F. solani*
R2A-7	*Sinomonas atrocyanea*	20.6 ± 0.038 ^d^	22.24 ± 0.018 ^e^	24.82 ± 0.028 ^d^	38.86 ± 0.018 ^e^	73.25 ± 0.030 ^a^	12.78 ± 0.021 ^d^	15.75 ± 0.038 ^cd^	8.93 ± 0.011 ^d^
KB-5	*Paenibacillus silvae*	17.35 ± 0.007 ^d^	27.16 ± 0.034 ^d^	19.48 ± 0.018 ^e^	51.90 ± 0.005 ^c^	33.55 ± 0.108 ^c^	18.00 ± 0.009 ^c^	8.34 ± 0.004 ^d^	17.85 ± 0.025 ^cd^
TSA-10E	*Priestia megaterium*	64.76 ± 0.003 ^b^	46.72 ± 0.024 ^a^	43.81 ± 0.019 ^a^	75.91 ± 0.024 ^a^	72.76 ± 0.034 ^a^	29.09 ± 0.006 ^b^	26.22 ± 0.010 ^ab^	52.29 ± 0.01 ^a^
TSA-1	*Bacillus pumilus*	46.27 ± 0.016 ^c^	34.30 ± 0.007 ^c^	36.61 ± 0.039 ^b^	66.23 ± 0.145 ^b^	35.18 ± 0.045 ^c^	26.83 ± 0.086 ^b^	12.93 ± 0.041 ^cd^	19.82 ± 0.015 ^bc^
TSA-6E	*Bacillus subtilis*	77.76 ± 0.016 ^a^	48.32 ± 0.018 ^a^	41.35 ± 0.028 ^a^	73.73 ± 0.041 ^a^	71.86 ± 0.015 ^a^	46.98 ± 0.018 ^a^	29.49 ± 0.055 ^a^	53.18 ± 0.020 ^a^
YG-2C	*Rossellomorea marisflavi*	59.69 ± 0.019 ^b^	37.48 ± 0.036 ^bc^	30.32 ± 0.043 ^c^	50.36 ± 0.038 ^cd^	57.01 ± 0.046 ^b^	29.50 ± 0.023 ^b^	19.52 ± 0.057 ^bc^	15.16 ± 0.081 ^cd^
KB-6	*Bacillus cereus*	55.90 ± 0.050 ^bc^	40.54 ± 0.069 ^b^	36.06 ± 0.008 ^b^	44.55 ± 0.080 ^de^	64.17 ± 0.032 ^ab^	26.00 ± 0.007 ^b^	20.87 ± 0.025 ^bc^	15.60 ± 0.100 ^cd^

Each value is the mean (±SE) of at least three replications. Values in columns followed by the same superscript letters indicate similar significance according to the multiple Duncan test (*p* < 0.05).

## Data Availability

The original contributions presented in this study are included in the article/Supplementary Material. Further inquiries can be directed to the corresponding authors.

## Supplementary Materials

The following supporting information can be downloaded at: https://www.mdpi.com/article/10.3390/biology14080921/s1, Table S1: Formulation of medium used; Table S2: Identification of fungal pathogens; Table S3: The rhizosphere microbes isolated on six different mediums.
