# Supplementary material for: Biocontrol Potential of Rhizosphere Bacteria Against Fusarium Root Rot in Cowpea: Suppression of Mycelial Growth and Conidial Germination
Source: Biology (Basel). 2025 Jul 23;14(8):921. doi: 10.3390/biology14080921 (PMC12384014; doi:10.3390/biology14080921)
Supplement: Supplementary file 1 [file biology-14-00921-s001.zip › biology-3683546-supplementary.pdf]

Table S1 Formulation of medium used

Medium name	Medium formulation (/L)
LB	10 g tryptone, 10 g NaCl, 5g yeast extract, 20 g agar, pH=7.2
0.1*LB	1 g tryptone, 1 g NaCl, 0.5g yeast extract, 20 g agar, pH=7.2
KB	20 g tryptone, KH2PO4 1.5 g, MgSO4 1.5 g, agar 10 g, pH=7.2-7.4
YG	5 g tryptone, 3 g yeast extract, 5 g glucose, 20 g agar, pH=7.2
TSA	solarbio, T8650-250g
R2A	solarbio, LA0700-250g
5*YEG	5 g yeast extract, 10 g glucose

Table S2 Identification of fungal pathogens

Isolate	Primer name	Amplicon size (bp)	Similarity (%)	Identification result
BEAN-1	TEF-1F/TEF-2R	825	97.67	*F. falciforme*
	TEF-1F/TEF-3R	900	99.40	
	EF-LF1/EF-LR1	1173	97.98	
	EF-LF1/EF-LR2	1217	95.88	
BEAN-3	TEF-1F/TEF-2R	813	98.87	*F. incarnatum*
	TEF-1F/TEF-3R	872	98.42	
	EF-LF1/EF-LR1	1175	96.31	
	EF-LF1/EF-LR2	1216	98.76	
BEAN-4	TEF-1F/TEF-2R	814	98.87	*F. oxysporum*
	TEF-1F/TEF-3R	870	97.92	
	EF-LF1/EF-LR1	1172	97.82	
	EF-LF1/EF-LR2	1220	98.06	

Table S3 The rhizosphere microbes isolated on six different mediums

Medium	Total of species	Species	The total number of microbial communities (cfu/ml)
TSA	8	*Bacillus aryabhattai*<br>*Bacillus mycoides*<br>*Bacillus pumilus*<br>*Bacillus subtilis*<br>*Fictibacillus barbaricus*<br>*Neobacillus ginsengisoli*<br>*Priestia aryabhattai*<br>*Priestia megaterium*	2.63E+07
R2A	11	*Bacillus bataviensis*<br>*Bacillus bingmayongensis*<br>*Bacillus ferrooxidans*<br>*Bacillus niacini*<br>*Bacillus pseudomycoides*<br>*Bacillus safensis*<br>*Dyella thiooxydans*<br>*Paenibacillus septentrionalis*<br>*Priestia aryabhattai*<br>*Priestia megaterium*<br>*Sinomonas atrocyanea*	3.03E+07
LB	13	*Bacillus aryabhattai*<br>*Bacillus ferrooxidans*<br>*Bacillus mycoides*<br>*Bacillus sporothermodurans*<br>*Falsibacillus pallidus*<br>*Neobacillus drentensis*<br>*Neobacillus ginsengisoli*<br>*Paenibacillus pabuli*<br>*Priestia aryabhattai*<br>*Priestia megaterium*<br>*Ralstonia pickettii*	2.73E+07

		Streptomyces geysiriensis<br>*Streptomyces triostinicus*	
KB	7	*Bacillus cereus*<br>*Fictibacillus barbaricus*<br>*Neobacillus citreus*<br>*Neobacillus ginsengisoli*<br>*Paenibacillus silvae*<br>*Priestia aryabhattai*<br>*Priestia megaterium*	5.13E+07
0.1LB	6	*Gottfriedia acidiceleris*<br>*Neobacillus niacini*<br>*Paenibacillus cellulositrophicus*<br>*Priestia megaterium*<br>*Streptomyces anandii*<br>*Trinickia diaoshuihuensis*	3.56E+07
YG	11	*Bacillus anthracis*<br>*Bacillus aryabhattai*<br>*Bacillus ferrooxidans*<br>*Bacillus pseudomycoides*<br>*Bacillus zanthoxyli*<br>*Fictibacillus barbaricus*<br>*Heyndrickxia oleronia*<br>*Neobacillus ginsengisoli*<br>*Priestia aryabhattai*<br>*Priestia megaterium*<br>*Rossellomorea marisflavi*	2.16E+07
